# Epigenetic reprogramming and re-differentiation of a Ewing sarcoma cell line

**DOI:** 10.3389/fcell.2015.00015

**Published:** 2015-03-09

**Authors:** Joseph B. Moore, David M. Loeb, Kyung U. Hong, Poul H. Sorensen, Timothy J. Triche, David W. Lee, Michael I. Barbato, Robert J. Arceci

**Affiliations:** ^1^Oncology, Sidney Kimmel Comprehensive Cancer Center, Johns Hopkins UniversityBaltimore, MD, USA; ^2^Department of Medicine, Institute of Molecular Cardiology, University of LouisvilleLouisville, KY, USA; ^3^Molecular Oncology, BC Cancer Research Centre, University of British ColumbiaVancouver, BC, Canada; ^4^Department of Pathology, Children's Hospital of Los AngelesLos Angeles, CA, USA; ^5^Ron Matricaria Institute of Molecular Medicine, Phoenix Children's Hospital, University of ArizonaPhoenix, AZ, USA; ^6^Medicine, Jefferson Medical CollegePhiladelphia, PA, USA

**Keywords:** Ewing sarcoma, induced pluripotent stem cells, reprogramming, epigenetics, drug resistance

## Abstract

Developmental reprogramming techniques have been used to generate induced pluripotent stem (iPS) cells from both normal and malignant cells. The derivation of iPS cells from cancer has the potential to provide a unique scientific tool to overcome challenges associated with the establishment of cell lines from primary patient samples and a readily expandable source of cells that may be used to model the initial disease. In the current study we developmentally reprogrammed a metastatic Ewing sarcoma (EWS) cell line to a meta-stable embryonic stem (ES)-like state sharing molecular and phenotypic features with previously established ES and iPS cell lines. EWS-iPS cells exhibited a pronounced drug resistant phenotype despite persistent expression of the oncogenic EWS-FLI1 fusion transcript. This included resistance to compounds that specifically target downstream effector pathways of EWS-FLI1, such as MAPK/ERK and PI3K/AKT, which play an important role in EWS pathogenesis. EWS-iPS cells displayed tumor initiation abilities *in vivo* and formed tumors exhibiting characteristic Ewing histopathology. In parallel, EWS-iPS cells re-differentiated *in vitro* recovered sensitivity to molecularly targeted chemotherapeutic agents, which reiterated pathophysiological features of the cells from which they were derived. These data suggest that EWS-iPS cells may provide an expandable disease model that could be used to investigate processes modulating oncogenesis, metastasis, and chemotherapeutic resistance in EWS.

## Introduction

Ewing sarcoma (EWS) is the second most common type of primary bone malignancy in children and adolescents (Ries and SEER Program (National Cancer Institute (US)), 1999; Burchill, 2003). EWS is a highly invasive, undifferentiated tumor of unknown histogenic origin whose molecular underpinnings, in approximately 85% of tumors, are associated with the expression of the EWS-FLI1 fusion product generated by the t(11;22)(q24;q12) chromosomal translocation (Arvand and Denny, 2001). The EWS-FLI1 transcript encodes a rogue transcription factor that deregulates the expression of various target genes, which is suggested to promote the activation of multiple pro-oncogenic signals (Lessnick and Ladanyi, 2012). Although EWS typically responds to initial combination chemotherapy treatment, approximately one-third of patients will suffer from recurrent, metastatic disease despite aggressive multimodal therapeutic approaches (Grier et al., 2003; Miser et al., 2007). Accordingly, the derivation of novel molecularly targeted therapies is paramount in improving patient outcomes and will require a greater understanding of the molecular mechanisms driving EWS oncogenesis, metastasis, and drug resistance. Although our principal understanding of the disease has advanced throughout the years with investigation of various EWS disease models, such approaches have presented with a number of limitations. As the cell of origin in EWS is unknown, a number of studies have depended on heterologous cell types as a model system for EWS to investigate the oncogenic fusion protein (May et al., 1993a,b, 1997); however, such model systems were later shown to lack the expression patterns of patient-derived tumors (Braunreiter et al., 2006; Hancock and Lessnick, 2008). Further, while the development of human model systems expressing EWS-FLI1 in various cell types more closely resemble gene expression alterations in EWS (Lessnick et al., 2002; Rorie et al., 2004; Hu-Lieskovan et al., 2005; Riggi et al., 2008), they do not accurately reproduce multiple aspects of the original disease. From a research standpoint, the identification of novel oncogenic mechanisms and therapeutic targets would ideally involve the use of patient-derived tumors, but this is complicated by inherent difficulties in establishing and maintaining primary tumor cell cultures. For instance, various primary EWS (Vormoor et al., 2001) as well as other established tissue sarcoma cell lines (Mills et al., 2009) simply fail to grow or exhibit slow growth kinetics *in vitro* which hamper their utility for scientific or clinical investigations. Moreover, those exhibiting slow growth phenotypes are likely more susceptible to the accrual of additional mutations and phenotypic alterations due to extended *in vitro* expansion times. Efforts to circumvent inherent issues associated with *in vitro* culture have involved the propagation of human primary tumor cells in murine xenograft models; however, these too have been met with various challenges regarding phenotypic preservation and patient tumor model accuracy. As is the case with a majority of human solid tumor xenograft models, the growth characteristics and tumor progression of xenotransplanted EWS cells (Scotlandi et al., 1998) unsuccessfully recapitulates the growth characteristics observed in patients and exhibits little histopathological resemblance to that of the original tumor from which the cells were derived (Mills et al., 2009). This highlights a current unmet need to identify additional *ex vivo* tumor cell propagation strategies that are focused toward the preservation of the molecular and phenotypic characteristics pathognomonic of the original diagnosed tumor.

Developmental reprogramming techniques have been used to generate iPS (induced pluripotent stem) cells from both normal (Takahashi et al., 2007; Park et al., 2008) and malignant cells (Utikal et al., 2009; Carette et al., 2010; Miyoshi et al., 2010; Kumano et al., 2012); a process that is achieved through the cellular transduction of a defined set of pluripotency transcription factors. This technology affords not only a unique scientific tool that may be utilized in the development of patient-specific stem cell-based regenerative therapies, but also in the establishment of disease models to investigate pathogenesis. Kumano et al. reported the successful derivation of iPS cells from primary chronic myelogenous leukemia (CML) patient samples (Kumano et al., 2012). These CML-derived iPS cells maintained expression of the oncogenic BCR-ABL fusion transcript (encoding a constitutively active, mutant tyrosine kinase), yet exhibited resistance to the receptor tyrosine kinase inhibitor, imatinib. Intriguingly, CML-iPS cells were capable of effectively re-differentiating into hematopoietic cells that recuperated sensitivity to imatinib, which reiterated pathophysiological features of the initial disease (Kumano et al., 2012). Such studies demonstrated that developmental reprogramming techniques may be employed to expand primary hematologic malignancies difficult to propagate *in vitro*. Further, Kumano and colleagues revealed that these iPS cells could be expanded *in vitro* without restriction and redifferentiated into CML hematopoietic cells that phenocopy the initial disease. This strategy affords the means to preserve the primary tumor phenotype and the ability to obtain a large quantity of viable cells that would be required for epigenomic, transcriptomic, proteomic, and importantly, large scale drug screen studies. Thus, we postulated that this technology may be extended to aid the investigation of other malignancies, including that of EWS, proven difficult to establish, maintain, and expand in culture. Therefore, once reprogrammed, EWS-iPS cells may provide an easily expandable and unlimited source of viable EWS cells that may be routinely obtained through their re-differentiation *in vitro*.

Given that some malignancies are refractory to iPS cell generation, here we sought to initially investigate whether or not a EWS cell line was susceptible to reprogramming and if re-differentiation of such cells could reproduce pathophysiological features of cells from which they were derived. In this study we developmentally reprogrammed EWS cells to a meta-stable ES-like state sharing a multitude of molecular features with previously established ES and iPS cell lines. EWS-iPS cells showed sustained expression of the EWS-FLI1 fusion transcript, yet were resistant to small molecule inhibitors of oncogenic pathways downstream of EWS-FLI1. Re-differentiated EWS-iPS maintained tumor formation competency *in vivo* giving rise to tumors with characteristic Ewing histopathology and demonstrated recovery of drug sensitivity upon re-differentiation *in vitro*, thus reiterating the pathophysiological features of the initial cell line.

## Materials and methods

### Cell culture

HEK293T cells were maintained in DMEM containing 10% FBS, 1% L-glutamine, and 1% penicillin/streptomycin. CHLA-10 cells (May et al., 2013) were grown in IMDM containing 20% FBS, 0.2% L-glutamine, and 1X ITS Premix (BD Biosciences). HEK293T and CHLA-10 cells were passaged every 4–5 days using trypsin or Puck's saline A solution, respectively. iPS cell lines were grown on mitomycin C-treated MEF feeder layers as previously described (Hotta et al., 2009) and maintained in ES media consisting of Knockout DMEM (Invitrogen) supplemented with 20% Knockout Serum Replacement (Invitrogen), 1X Nonessential amino acids (Invitrogen), 1X Glutamax (Invitrogen), 1X Antibiotic/Antimycotic (Invitrogen), 0.1 mM 2-mercaptoethanol (Invitrogen), 10 ng/ml human bFGF (Invitrogen), and 1 μg/ml puromycin. ES media was replaced daily and iPS cells were transferred to newly derived MEF feeder layers every 5 days following collagenase IV dissociation (1 mg/ml for 20 min at 37°C), as previously described Hotta et al., 2009.

### iPS cell line derivation

Cellular reprogramming was performed using a variation of a previously described method (Yu et al., 2009). CHLA-10 or HEK293T cells were induced to pluripotency via nucleofection-mediated delivery of an oriP/EBNA1-based episomal vector (pEP4-EO2S-EN2K: Addgene 20925, Yu et al., 2009) containing OCT4, SOX2, NANOG, and KLF4 together with a reporter plasmid [PL-Sin-EOS-S(4+)-EiP: Addgene 21314 (Hotta et al., 2009)]. The reporter construct contained eGFP and puromycin selectable markers under the control of a Sox2-responsive ETn promoter. Sample preparation and Nucleofection was performed using the Neon^™^ Transfection system (Invitrogen) as directed by the manufacturer's instructions. Briefly, two million CHLA-10 or HEK293T cells were electroporated with 5 μg of reporter with either 5 μg of reprogramming or empty vector using a single 40 ms pulse of 1100 volts. Electroporated cells were plated on MEF feeder layers maintained in DMEM complete media lacking antibiotics. Four days following electroporation, DMEM complete media was replaced with ES complete media. Thirteen days after nucleofection, cells were transferred to fresh ES media containing 1 μg/ml puromycin. Approximately 1 month following transduction of the reprogramming factors, cultures were screened for the emergence of iPS cell colonies.

### Immunocytochemistry

iPS cell colonies grown were fixed in 4% paraformaldehyde, permeabilized with 0.25% Triton X-100, and blocked in 5% goat serum in PBST. Cells were incubated with primary antibody overnight at 4°C, washed with PBST, and incubated with DyLight 650 (ab98371 or ab102464; Abcam) conjugated secondary antibody. Rat monoclonal to SSEA3 (ab16286), mouse monoclonal to SSEA4 (ab16287), and mouse monoclonal to TRA-1-60 (ab16288) antibodies were supplied by Abcam, in addition to rat IgM (ab35768) and mouse IgM (ab18401) isotype controls. iPS cells were counter stained with 0.5 μg/ml DAPI and rinsed with PBS prior to fluorescence microscopic imaging.

### PCR analysis of OriP/EBNA1-based episomal vector

Episomal plasmid DNA was purified from iPS cells as performed by Ziegler et al. (2004) and assessed for the presence of vector specific OCT4, SOX2, NANOG, and KLF4 transgenic sequences via polymerase chain reaction with specified primers as formerly described by Yu et al. (2009). Purified pEP4 backbone/empty vector and pEP4-EO2S-EN2K reprogramming plasmid served as negative and positive controls in PCR reactions, respectively.

### Semi-quantitative RT-PCR analysis of pluripotency genes

Total RNA was isolated and prepared using TRIzol® reagent (Invitrogen), as per manufacturer instructions. 500 ng of total RNA was reverse transcribed using the Bio-Rad iScript^™^ cDNA Synthesis Kit (Bio-Rad) with Oligo(dT) primers. Semi-quantitative PCR was performed to assess the RNA expression of total, endogenous and recombinant pluripotency genes. The primers used for detection of total NANOG, OCT4, SOX2, and KLF4 are as follows: Nanog_t_ primers, 5′-GATTTGTGGGCCTGAAGAAA-3′ and 5′-AAGTGGGTTGTTTGCCTTTG-3′; Oct4_t_ primers, 5′-CTCACCCTGGGGGTTCTATT-3′ and 5′-AGCTTCCTCCACCCACTTCT-3′; Sox2_t_ primers, 5′-CAAGAC GCTCATGAAGAAGG-3′ and 5′-GTTCATGTGCGCGTAACTGT-3′; Klf4_t_ primers, 5′-ACCCACACAGGTGAGAAACC-3′ and 5′-ATGTGTAAGGCGAGGTGGTC-3′. The human actin gene was detected as an endogenous control with the following primers: Actin (forward): 5′-GACCTGGCTGGCCGGGACCT-3′, Actin (reverse): 5′-GGCCATCTCTTGCTCGAAGT-3′. PCR was performed using recombinant, Taq DNA Polymerase (Invitrogen) according to the manufacturer's protocol for 35 cycles with an annealing temperature of 62°C. Amplified products were resolved on 1X TBE agarose gels and visualized by ethidium bromide staining.

### Alkaline phosphatase staining

iPS cell colonies were cultured on MEF feeder layers for 5 days. Cells were then analyzed for AP activity using an Alkaline Phosphatase Detection Kit (SCR004; Millipore, Billerica, MA) according to the manufacturer's protocol.

### *In vitro* embryoid body formation

To induce embryoid body formation, iPS cells were dissociated from MEF feeder layers with collagenase IV, transferred to plastic Petri dishes containing ES medium without bFGF, and cultured in suspension for 6 days.

### *In vivo* teratoma and tumor formation assays

iPS cell colonies were dissociated (1 mg/ml collagenase IV) from MEF feeder layers, washed with DMEM base media, and suspended in DMEM containing 10% FBS to an approximate final concentration of 2 × 10^7^ cells/ml. Resultant colonies, consisting of approximately 1–2 million cells (100 μL), were subcutaneously injected into 5–7 week old NOG (NOD/Shi-scid/IL-2Rγ^null^) mice. Injected mice were monitored for 6–12 weeks and tumor dimensions measured with precision calipers. Tumors approaching 0.75 cm^3^ in size were excised, rinsed with PBS, and fixed in 10% formalin. Hematoxylin/Eosin staining was performed by the Department of Molecular and Comparative Pathobiology at the Johns Hopkins School of Medicine. For tumor formation assays EWS-iPS cells were dissociated from feeder layers, mechanically separated into individual cell suspensions to promote differentiation, and suspended in IMDM base media containing 20% FBS, 0.2% L-Glut, 0.1% ITS prior to subcutaneous injection (approximately 1 million cells). Subsequently, mice were monitored for 6–12 weeks and tumor dimensions measured. Tumor growth rates ($\frac{\Delta Log(tumor\;volume(mm^{3})}{\Delta \;Time\;(days)}$) were determined and reported using linear regression analysis of semi-log plots {Log [tumor volume (mm^3^)] vs. Time (days)}. Again, tumors reaching 0.75 cm^3^ in size were excised, rinsed with PBS, and fixed in 10% formalin. Hematoxylin/Eosin staining was performed by the Molecular and Comparative Pathobiology core.

### Chemo-sensitivity assays

Approximately 5000 CHLA-10 parent or EWS-iPS cells were plated per well of 0.1% porcine gelatin coated 96-well tissue-culture plates in triplicate. The following day, parental and EWS-iPS cell medium was replaced with 100 μL of 10 μM YK-4-279, 0.1 μM mithramycin, 300 μM U-0126, 30 μM rapamycin, 100 μM LY294002 or untreated DMSO vehicle in ES media and incubated for 48 h. During drug exposure, all cultures were incubated in iPS cell base media containing 10 ng/ml human bFGF to control for potential variations in drug resistance due to growth factor signaling (Dini et al., 2002). Cell viability was subsequently assessed using a Cell Titer 96® Aqueous One Solution Cell Proliferation Assay (MTS Reagent; Promega) according to the manufacturer's instructions. Drug responses were determined for all cell lines by comparing the amount of formazan product formation in treated to untreated cells by measuring spectrophotometric absorbance at 490 nanometers. For chemo-sensitivity assays utilizing *in vitro* differentiated cells, EWS-iPS were manually dissociated into individual cell suspensions and plated in Ewing complete media in the absence of bFGF for a period of 48 h prior to drug exposure.

### DNA methylation profiling

Genomic DNA was extracted using the Wizard® Genomic DNA Purification Kit (Promega) and 300 ng bisulfite converted using the methylSEQr^™^ Reagent Kit (Applied Biosystems) according to each manufacturer's protocol. Bisulfite converted DNA was hybridized to an Infinium 450K Bead Chip (Illumina). The 450K chip was processed and scanned by the Sydney Kimmel Comprehensive Cancer Center Microarray Core facility at the Johns Hopkins School of Medicine. 450K data was background subtracted and filtered of null-signal probes in Genome Studio and subsequently exported to R version 2.15.1 × 64 bit (The R Project for Statistical Computing). The level of methylation at each CpG site, defined as the ratio of methylated allele to the sum of methylated and unmethylated alleles, or β-value, ranges from 0.0 to 1.0 (completely unmethylated to completely methylated, respectively). β-values were analyzed within R utilizing the Bioconductor (version 2.5) and Methylumi (version 2.2.0) statistical packages. Unsupervised hierarchical clustering was used to examine methylation status in an unbiased fashion, and clustering was performed, with Euclidian distance and complete linkage using the heatmap.2 function in the gplots library.

### Accession numbers

Previously published 450K DNA Methylation array data from 25 ES, 29 iPS, 6 differentiated ES, 6 differentiated iPS cells, and 5 hydatidiform mole samples are available at the NCBI GEO database under the accession series designation GSE31848.

### EWS-FLI1 gene expression analysis

Total RNA was isolated and prepared using TRIzol® reagent (Invitrogen) according to the manufacturer's instruction. 500 ng of total RNA was reverse transcribed using a Bio-Rad iScript^™^ cDNA Synthesis Kit (Bio-Rad, Hercules, CA) with random primers. Quantitative PCR was performed with iQ^™^SYBR® Green Supermix (Bio-Rad) in a Bio-Rad CFX96 Real-Time Instrument. The primers used for the detection of the EWS-FLI1 fusion transcript included the following: EWS-FLI1 (forward): 5′-TCCTACAGCCAAGCTCCAAGTC-3′ and EWS-FLI1 (reverse): 5′-GAATTGCCACAGCTGGATCTGC-3′. The human porphobilinogen deaminase (PBGD) gene was detected as an internal control for normalization with the following primers: PBGD (forward): 5′-GGAGCCATGTCTGGTAACGGCA-3′, PBGD (reverse): 5′-GGTACCCACGCGAATCACTCTCA-3′). PCR was performed with an initial denaturation at 95°C for 3 min, followed by 35 cycles of 30 s at 95°C, 30 s at 55°C, and 30 s at 72°C. Relative gene expression was calculated according to the ΔΔC_T_ method for quantitative real-time PCR.

### Statistical analysis

Statistical analyses were performed using the descriptive statistics data analysis toolpak add-in available through the Microsoft Office Excel program. Statistical significance (*p*-values) among data sets was determined using a student's two-tailed *t*-test.

## Results

### Developmental reprogramming of the metastatic EWS cell line, CHLA-10

**Figure 1:**
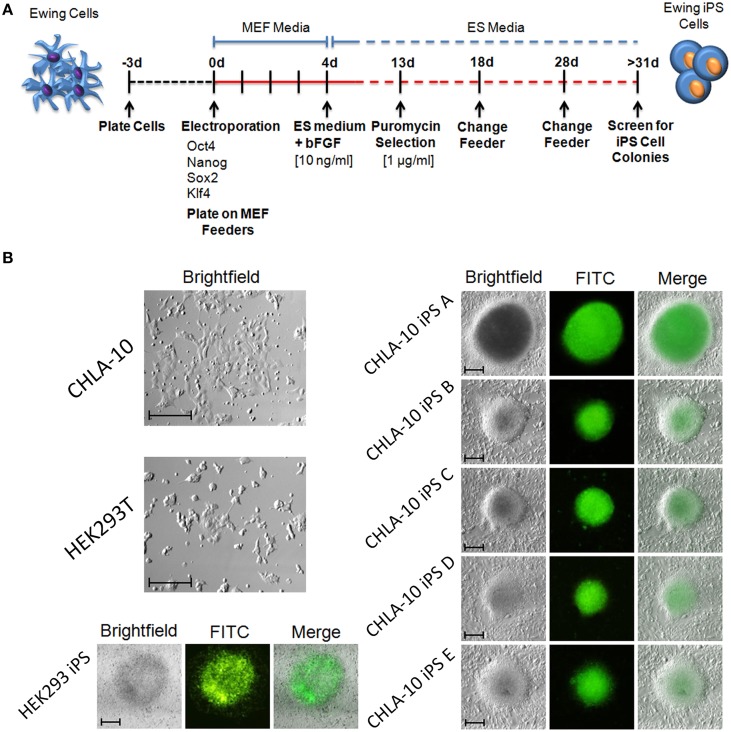
**Generation of iPS cells from the Ewing cell line, CHLA-10**. **(A)** Cell reprogramming schematic and timeline. **(B)** EWS-iPS cell colonies (A–E) enriched for the expression of SOX2 via puromycin selection are discernible by GFP fluorescence. Parent (HEK293T and CHLA-10), as well as HEK293T iPS cell controls are shown (scale = 200 micron).

**Figure 2:**
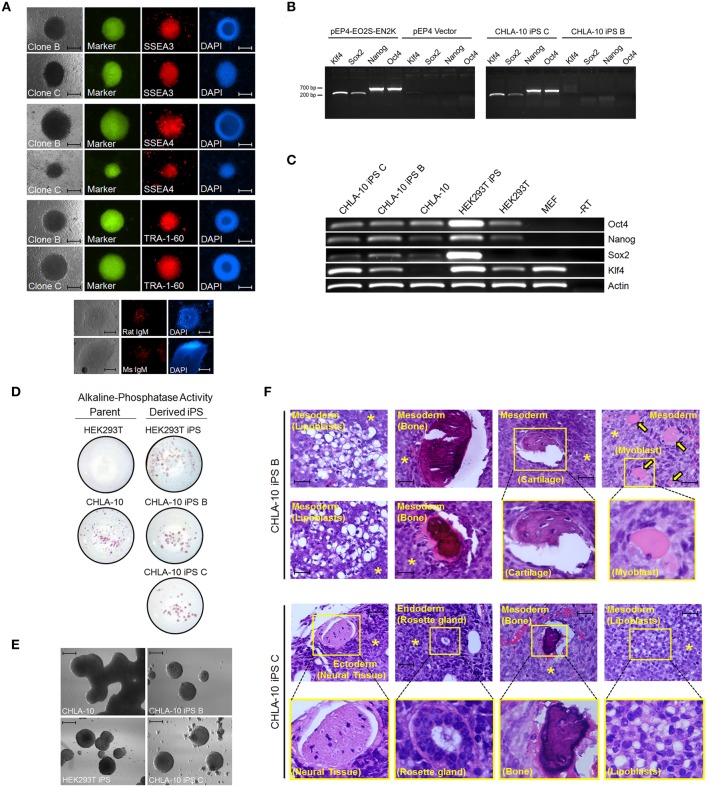
**EWS-iPS cell characterization**. **(A)** Immunofluorescence microscopic detection of stem cell specific antigens (SSEA3, SSEA4, and TRA-1-60) in EWS-iPS clones. Brightfield, FITC, and DAPI nuclear stained cells are presented. Mouse IgM and rat IgM negative controls are shown. Scale = 200 micron. **(B)** PCR analysis of the EBNA1/oriP-based transgenic reprogramming factors in EWS-iPS clone B and clone C cells (right panel). Purified pEP4-EO2S-EN2K and empty pEP4 reprogramming plasmid controls are shown (left panel). **(C)** RT-PCR analysis of total ES cell-specific transcripts. Reverse transcriptase minus (-RT) control reactions are shown. **(D)** Alkaline phosphatase staining of parent (left column) and derived iPS cells (right column). **(E)** *In vitro* embryoid body (EB) formation assays. CHLA-10 parent, EWS-iPS B and C, and control HEK293T-iPS EBs. Scale = 200 micron. **(F)** Teratoma formation assays. H&E sections of control CHLA-10 (top panel), EWS-iPS cell clone B (middle panels), and EWS-iPS cell clone C (bottom panels) xenografts are shown. Astericks (^*^) denote undifferentiated Ewing cells and yellow arrows denote myoblasts. Magnified images are depicted in yellow boxes. Scale = 200 micron.

In the current study we sought to developmentally reprogram an established metastatic EWS cell line sourced from a patient-derived primitive neuroectodermal tumor (PNET), designated CHLA-10 (May et al., 2013), via nucleofection-mediated delivery of an EBNA1-based 4 factor (OCT3/4, NANOG, SOX2, and KLF4) episomal construct (Figure 1A). This particular cell line was considered ideal for preliminary reprogramming studies due to its uniform phenotype, relatively short doubling time (≈32 h), and amenability to electroporation. Utilizing the aforementioned strategy, putative reprogrammed CHLA-10 or EWS-iPS colonies were generated. They exhibited classical iPS cell morphological features and closely resembled HEK293T iPS cells generated by the same reprogramming strategy (Figure 1B). Putative EWS-iPS cell colonies were distinguished from partially reprogrammed cells by the presence of stem cell-specific antigens. Of the suspected iPS cell clonal populations isolated, immunofluorescence spectroscopy confirmed clones B and C to express Tra-1-60, SSEA-3 and SSEA-4 stem cell markers (Figure 2A). Using these clones, two EWS-iPS cell lines free of integrated transgenic sequences were generated. OriP/EBNA-based episomal vectors are gradually lost through mitosis during clonal expansion (Yu et al., 2009; Chou et al., 2011). Consistent with these observations, PCR analysis of the transgenic reprogramming factors demonstrated the high passage EWS-iPS clone B (>25 passages) to have lost and the low passage EWS-iPS clone C (<10 passages) to have maintained the episomal plasmid (Figure 2B). Both clones exhibited an increase in total expression levels (endogenous and transgenic sources) of characteristic embryonic stem cell transcripts (OCT3/4, NANOG, SOX2, and KLF4), as evidenced by semi-quantitative PCR (Figure 2C). In addition, alkaline phosphatase (AP) activity was measured to evaluate the undifferentiated state of EWS-iPS cells. Relative to the HEK293T iPS cell positive control line, both EWS-iPS clones B and C also expressed substantial levels of AP (Figure 2D). However, high levels of AP activity observed in the CHLA-10 parent strain precluded the utility of this approach as a distinctive marker.

The differentiation potential of EWS-iPS cells was subsequently assessed by performing an embryoid body formation assay. To induce embryoid body formation, bFGF was withdrawn from the media and both EWS-iPS cells (clones B and C) and parental cells were grown in suspension on petri dishes for 6 days. Like the HEK293T iPS cell control, both EWS-iPS cell clones (B and C) displayed typical embryoid body features forming characteristic spheroid aggregates with increasingly complex interiors, indicative of cellular differentiation (Figure 2E). In contrast, this was not observed with CHLA-10 parental cells cultured under the same conditions where growth appeared unorganized.

Further, teratoma formation assays were performed to assess the differentiation potential of the EWS-iPS cell lines (Figure 2F). Tumors derived from EWS-iPS cell clone B exhibited a restricted capacity to spontaneously differentiate into the three embryonic germ lineages and predominately formed tissues of mesodermal origin, i.e., adipose, bone, cartilage, and skeletal muscle. EWS-iPS cell clone C exhibited a broader differentiation capacity and gave rise to all three embryonic lineages (endoderm, ectoderm and mesoderm), including cells of adipogenic and osteogenic lineage, as well as glandular and neural tissues. The disparity in differentiation capacity between these EWS-iPS cell lines demonstrates the variable developmental states between clones and further, may also reflect differences in iPS cell quality related to passage number (Okada and Yoneda, 2011). Despite the high developmental potential of both EWS-iPS cell lines, a moderate fraction of undifferentiated cells were visible in both sets of teratomas, indicating that these reprogrammed cells remain capable of contributing to poorly differentiated tumorigenic tissue exhibiting characteristic EWS histology (see below for further details).

### EWS-iPS cells share methylation patterns with other ES and iPS cell lines

**Figure 3:**
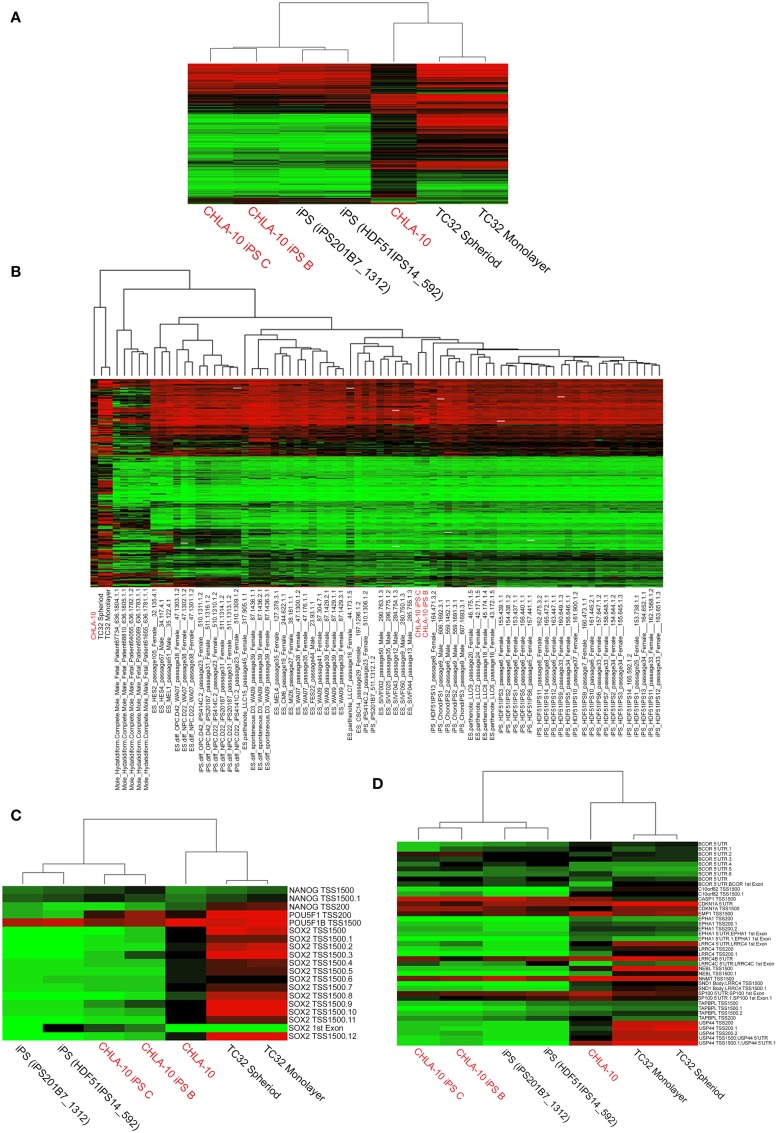
**Comprehensive analysis of DNA methylation. (A,B)** Dendograms depicting unsupervised hierarchical clustering based on differential methylation at 7687 differentially methylated CpGs. In the accompanying heatmaps, green indicates a CpG methylation of less than 50% and red of more than 50%. Unsupervised hierarchical clustering based on differential methylation at **(C)** promoter regions of endogenous ES pluripotency genes and **(D)** stem cell specific DMRs. Heatmaps depict background subtracted β-values. TSS and 5′UTR denote probes located at transcriptional start sites and 5′untranslated regions, respectively. Methylation values for comparative cell lines (25 ES, 6 differentiated ES, 29 iPS, 6 differentiated iPS, and 5 hydatidiform mole samples) were obtained from the NCBI GEO database.

An analysis of DNA methylation demonstrated comparable methylation patterns among EWS-iPS and 2 iPS cell lines obtained from the NCBI GEO database (Figure 3A). Further, these methylation patterns were distinctly different from both the CHLA-10 parent and TC32 Ewing cell lines grown as spheroids or in monolayer. A more extensive comparison of DNA methylation (Figure 3B) with additional cell lines and tissues revealed that EWS-iPS cells possessed methylation patterns that more closely resembled those of lower passaged ES and iPS cell lines and were distinct from the methylation patterns of the more differentiated tissues (differentiated ES and iPS cells, hydatidiform moles, the TC32 EWS cell line and the CHLA-10 Ewing parent cell lines). Moreover, multiple CpG sites spanning the promoter regions of some key endogenous ES pluripotency genes (i.e., *NANOG*, *POU5F1*, and *SOX2*) exhibited similar hypomethylated patterns among EWS-derived and other iPS cells (Figure 3C). Previous work investigating iPS cell methylation dynamics has aided in the identification of stem cell-specific densely methylated regions (DMRs) that are subject to significant expression alterations in human iPS cells (Nishino et al., 2011). Consistent with these reports, we identified numerous altered CpG sites in EWS-iPS cells (compared to parent Ewing cell lines), spanning the promoter regions of several stem cell specific DMRs, that demonstrated comparable methylation patterns with previously established iPS cells (Figure 3D).

### EWS-iPS cells maintain EWS-FLI1 oncogene expression yet acquire resistance to targeted agents

**Figure 4:**
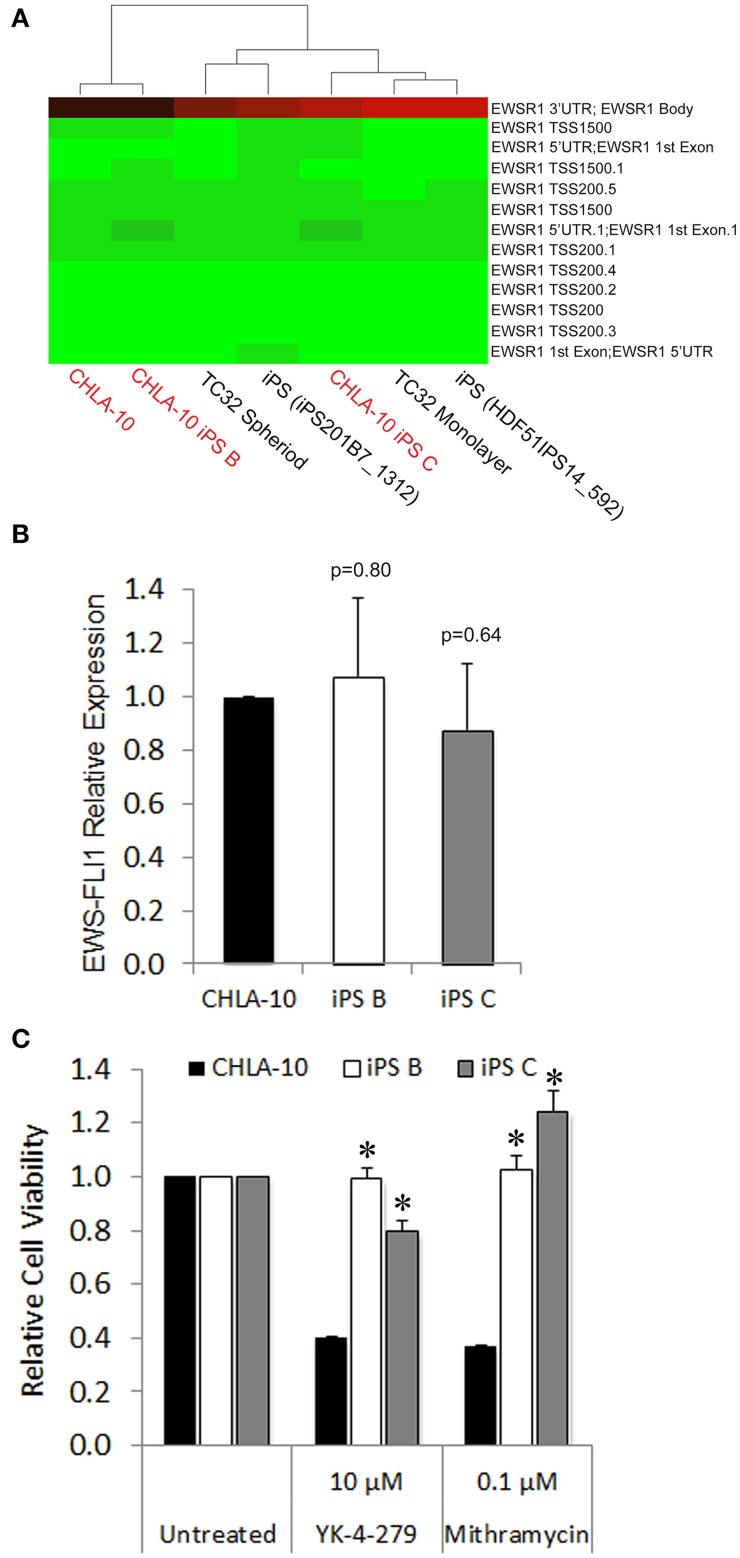
**EWS-iPS cells demonstrate preservation of oncogene expression and acquired resistance to EWS-FLI1 targeted agents**. **(A)** Dendograms depicting the methylation status of CpG sites spanning the EWS promoter of CHLA-10 parent, TC32 Ewing (grown in monolayer or as spheriods), EWS-iPS (clones B and C), and control iPS (iPS201B7_1312 and HDF51IPS_592) cell lines. Green indicates a CpG methylation of less than 50% and red of more than 50%. TSS and 5′UTR denote probes located at transcriptional start sites and 5′untranslated regions, respectively. **(B)** EWS-FLI1 gene expression in CHLA-10 parent (*n* = 4) and EWS-iPS (*n* = 4) (clones B and C) cells. Standard error of the mean is reported. *p* = 0.80 (iPS B vs. CHLA-10) and *p* = 0.64 (iPS C vs. CHLA-10). **(C)** Drug sensitivity assays. CHLA-10 parent (*n* = 6), iPS B (*n* = 3), or iPS C (*n* = 3) cell lines were exposed to chemotherapeutic agents or DMSO vehicle (untreated) in iPS base media containing 10 ng/ml human bFGF for a period of 48 h. Following treatment, MTS assays were performed and relative cell viabilities determined (relative cell viability = 490 nm absorbance treated/490 nm absorbance untreated). Standard error of the mean is reported. ^*^p < 0.0001 (iPS vs. CHLA-10).

In a previous study, iPS cells generated from CML patient-derived tumors exhibited an acquired resistance to receptor tyrosine kinase-targeted inhibition with imatinib despite unremitting expression of the BCR-ABL oncogene (Kumano et al., 2012). Such studies prompted us to investigate whether cellular reprogramming influenced the methylation status of the EWS promoter and/or altered corresponding oncogenic EWS-FLI1 transcript expression levels in EWS-derived iPS cells. In methylation array analyses EWS-iPS cells (clones B and C) exhibited hypomethylation marks at numerous CpG sites spanning the EWS promoter region and were comparable to that of the parental CHLA-10, the TC32 Ewing cell line (grown as spheroids or in monolayer), and two unrelated iPS cell lines (iPS201B7 and HDF51IPS14) (Figure 4A). Consistent with the observed EWS promoter hypomethylation, quantitative PCR revealed equivalent expression of the EWS-FLI1 fusion transcript among EWS-iPS and the CHLA-10 parental cells from which they were derived (Figure 4B), indicating that erasure of the epigenetic modifications during cellular reprogramming does not disrupt EWS-FLI1 oncogene expression in EWS-iPS cells. In light of this observation, we next determined if EWS-iPS and CHLA-10 parental cells would exhibit equivalent sensitivities to mithramycin or YK-4-279, both pharmacological agents that have been shown to effectively target and inhibit EWS-FLI1 activity (Grohar et al., 2011; Barber-Rotenberg et al., 2012). Cell lines were exposed to mithramycin, YK-4-279, or DMSO vehicle for a period of 48 h and MTS assays were subsequently performed to determine relative cell viabilities (Figure 4C). Interestingly, cell viability assays demonstrated that EWS-iPS cells are largely resistant to YK-4-279 and mithramycin relative to the CHLA-10 parental cell line (Figure 4C). Based on such data it appears that EWS-iPS cells have developed resistance to pharmacological EWS-FLI1 inhibitors in spite of persistent expression of the oncogenic fusion product. This suggests that epigenetic alterations imposed during the reprogramming process provided the EWS cells drug resistance.

### EWS-iPS cells exhibit tumor initiating properties

**Figure 5:**
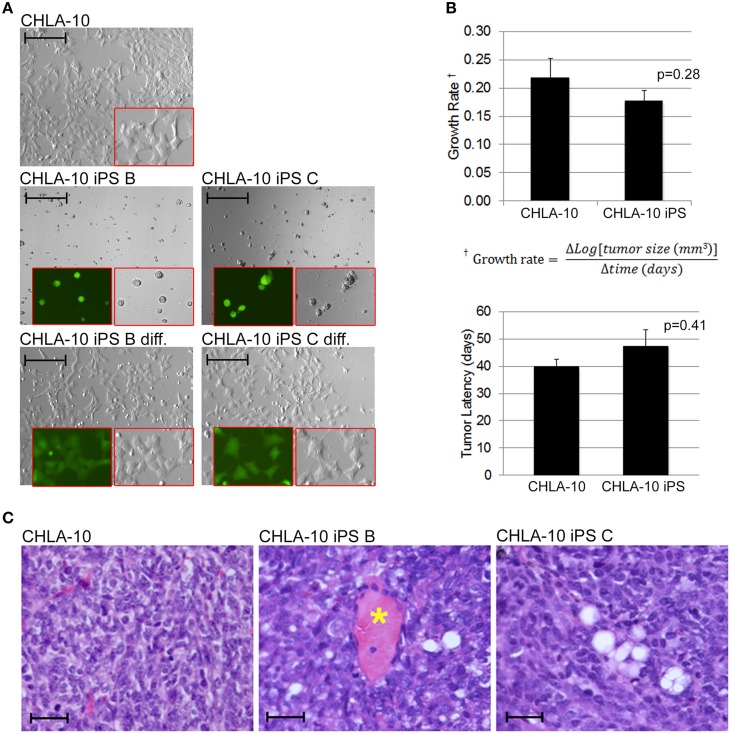
**EWS-iPS cells exhibit tumor initiating properties**. **(A)** EWS-iPS cells differentiate into Ewing cells that morphologically resemble CHLA-10 parental cell lines *in vitro*. EWS-iPS (clones B and C) were dissociated into individual cell suspensions (middle panels) and differentiated by plating in Ewing media [IMDM, 20% FBS, 0.2% L-Glut, 0.1% ITS] for a period of 48 h (bottom panels). Red boxes denote magnified fields. Brightfield or SOX2-dependent GFP marker channels are shown (scale = 200 micron). **(B)** *In vivo* differentiated EWS-iPS (*n* = 5) and parental (*n* = 9) cell lines possess comparable *in vivo* growth rates (*p* = 0.28; iPS vs. CHLA-10) and tumor latencies (*p* = 0.41; iPS vs. CHLA-10). Standard error of the mean is reported. **(C)** H&E stained sections of control CHLA-10 (left panel), EWS-iPS B (middle panel), and EWS-iPS C (right panel) tumor xenografts are shown. Astericks (^*^) denote blood vessels. Scale = 200 micron.

As the EWS-iPS cells appeared to have undergone significant phenotypic alterations during the reprogramming process, we sought to determine if these cells were competent to re-differentiate into EWS-like cells from which they were derived. *In vitro* differentiation was induced by plating individual cell suspensions in IMDM media for 48 h. EWS-iPS cells readily re-differentiated into cells that were morphologically indistinguishable from the parental CHLA-10 cells (Figure 5A). SOX2 expression was also shown to decrease upon *in vitro* differentiation of EWS-iPS cells, as demonstrated by a reduction in intensity of the SOX2-responsive green fluorescence reporter. Contrary to differentiated iPS, EWS-iPS cell suspensions cultured in ES medium displayed small-round-refractile morphology with sustained expression of the SOX2-responsive GFP reporter (Figure 5A). Next, to investigate the tumor initiation competency and engraftment potential of these cells we next assessed the *in vivo* growth rates and latencies of tumors that developed following subcutaneous injection of EWS-iPS or CHLA-10 parental cell lines into immune-compromised mice. Both parental and EWS-iPS cells exhibited comparable growth rates and tumor latencies (Figure 5B). Importantly, EWS-iPS cells readily formed undifferentiated tumor cells exhibiting the small-blue-round-cell morphology that is characteristic of EWS (Figure 5C). Thus, EWS-iPS cells exhibited comparable tumor initiating properties with similar growth kinetics to that of the CHLA-10 parental cells, were able to re-differentiate into EWS-like cells *in vitro*, and readily formed small-blue-round-cell tumors, characteristic of Ewing histopathology *in vivo*.

### Re-differentiated EWS-iPS cells recover sensitivity to agents targeting pathways downstream of EWS-FLI1

**Figure 6:**
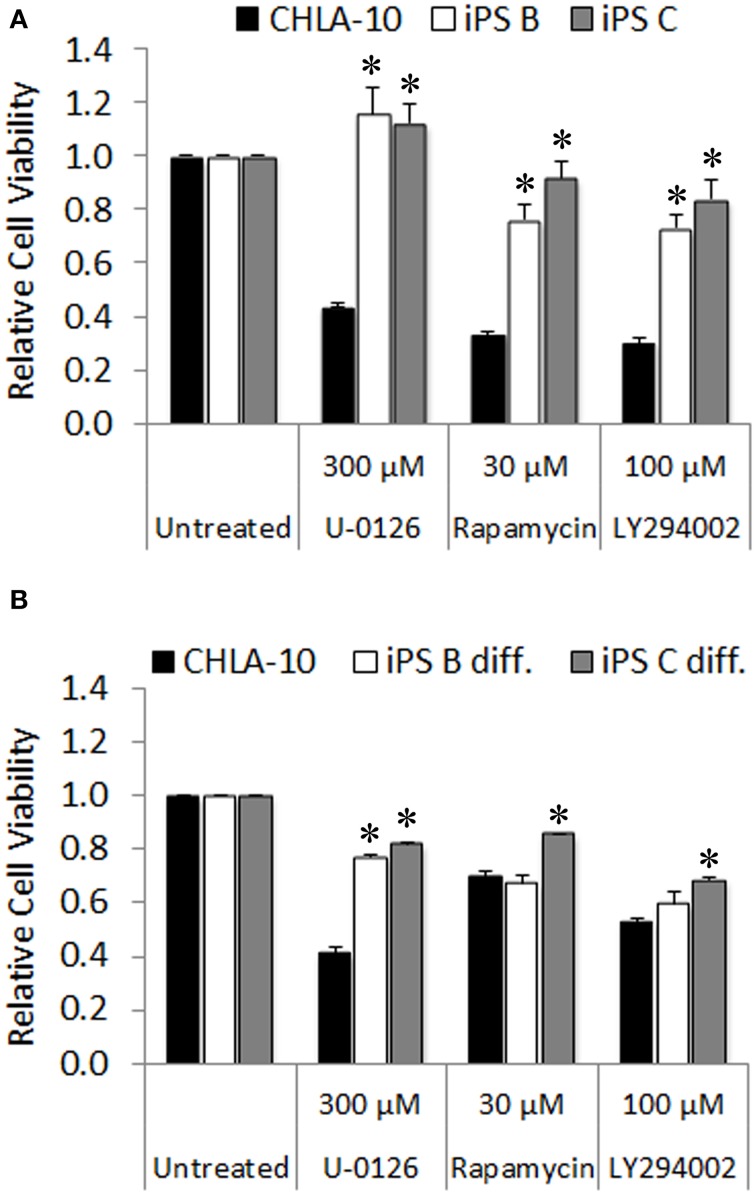
**Re-differentiated EWS-iPS cells recover sensitivity to chemotherapeutic agents targeting oncogenic pathways downstream of EWS-FLI1**. CHLA-10 parent (*n* = 6) and **(A)** iPS (clones B and C; *n* = 3) or **(B)** differentiated iPS (iPS diff. clones B and C; *n* = 3) were exposed to chemotherapeutic agents or DMSO vehicle (untreated) in iPS cell base media containing 10 ng/ml human bFGF for a period of 48 h. Following treatment, MTS assays were performed and relative cell viabilities determined (relative cell viability = 490 nm absorbance treated/490 nm absorbance untreated). Standard error of the mean is reported. ^*^p < 0.005 (iPS vs. CHLA-10).

Ewing cells characteristically form poorly differentiated tumors that lack distinguishing markers identifying mature tissue types, thus we additionally utilized *in vitro* chemotherapeutic sensitivity to agents targeting downstream effector pathways of EWS-FLI1 as a proxy for EWS differentiation of Ewing-derived iPS cells. Given that both the MAPK/ERK and PI3K/AKT/mTOR signaling cascades serve as major modulators of the oncogenic phenotype in both EWS and EWS-FLI1 expressing cancer cell lines (Silvany et al., 2000; Zenali et al., 2009), we chose U-0126, rapamycin, and LY294002 pharmacological agents (targeted inhibitors of ERK1/2, mTOR, and PI3K, respectively) for investigation in subsequent chemo-sensitivity studies. CHLA-10 parental cells exhibited significant sensitivity to the aforementioned molecularly targeted agents (Figure 6A). In contrast, EWS-iPS cells (clones B and C) were remarkably resistant to inhibitors targeting either the MAPK/ERK or PI3K/AKT pathways, suggesting a potential loss of dependency upon the activity of these pro-oncogenic signaling cascades downstream of EWS-FLI1 in reprogrammed cells. Further, we investigated whether re-differentiated EWS-iPS cells would recover sensitivity to other targeted agents. As shown in Figure 6B, *in vitro* differentiated iPS cells exhibited recuperated sensitivity to rapamycin and LY294002, inhibitors targeting mTOR and PI3K, respectively; an effect that was more pronounced in EWS-iPS clone B compared to clone C. In an analogous fashion, both differentiated iPS cell clones showed a similar degree of drug response recovery to the ERK inhibitor, U-0126; however the magnitude of reduction in cell viability of the iPS cells was less severe vis-à-vis CHLA-10 parental (Figure 6B). Hence, while undifferentiated EWS-iPS cells were resistant, re-differentiated EWS-iPS cells appeared to recuperate some sensitivity to inhibitors of putative downstream EWS-FLI1 effector pathways, similar to the original CHLA-10 cell line.

## Discussion

While cancer cell nuclei have been suggested to not be amenable to the reprogramming process (Hochedlinger et al., 2004), multiple groups have generated putative iPS cells from various types of malignancies (Carette et al., 2010; Miyoshi et al., 2010; Kumano et al., 2012); however, reprogramming efficiencies were reported to be low. In a synonymous manner, the derivation of iPS from EWS cells via the introduction of a 4-factor (OCT4, NANOG, SOX2, and KLF4) episomal construct yielded reduced reprogramming rates with an approximate efficiency of 0.0001%. Our EWS-iPS cells displayed characteristic iPS cell-like morphology, expressed ES cell surface markers, and exhibited DNA methylation patterns comparable to other ES and iPS cell lines. While hierarchical cluster analysis of our EWS-iPS cells demonstrated similar patterns of global methylation to other ES and iPS cell lines, the degree of promoter hypomethylation of several stem cell-specific DMRs and some endogenous pluripotency genes was not as pronounced in our EWS-iPS cells, thus suggesting that the EWS-derived iPS cells may have been only partially reprogrammed. This appeared to be the case in a recent study which attempted to reprogram a EWS cell line via employing a herpesvirus saimiri-based (HSV) 3 factor (OCT4, NANOG, and LIN28) vector; these cells, termed “induced multipotent cancer cells (iPCs),” yielded only a partially reprogrammed phenotype capable of differentiation toward the ectodermal lineage (Brown et al., 2013). Contrary to this report, our EWS-derived iPS cell clones (specifically B and C) exhibited capacity to differentiate into a number of embryonic tissues. Both clones demonstrated proclivity to differentiate into mesodermal tissues, and clone C exhibited potential to form additional tissues of both endodermal and ectodermal origin (although less frequently than that of mesoderm)—suggesting achievement of a true pluripotent state. The disparity in differentiation capacity between these clones may reflect the acquisition of differing epigenetic states as a result of the inherent stochastic nature of the reprogramming process. However, despite the fact that the reprogrammed EWS cell-derived clones exhibit many of the prototypical iPS/ES cell markers and phenotypic features, they fail to fully recapitulate the developmental potential of other well established iPS cell lines generated from non-malignant tissues (Takahashi et al., 2007). Whether this is a consequence of deficiencies in the reprogramming method employed in their derivation or oncogene-mediated blockade of the proper coordinated epigenetic alterations required for successful reprogramming is currently unknown. However, maintenance of specific epigenetic marks, or rather maintaining some “epigenetic memory” of the original EWS cell line, could prove advantageous for the purpose of accurately modeling a particular disease following successful re-differentiation.

Indubitably, numerous preclinical models have aided in improving our understanding of the molecular basis of EWS. However, the elucidation of novel molecular mechanisms driving oncogenesis and metastasis, as well as the identification of new effective therapies for EWS has been hindered by inherent limitations in existing tumor cell models. These include studies utilizing established cell lines that have been extensively passaged *in vitro*, as well as those employing the transplantation of primary tumor cells into tumor xenografts models (Mills et al., 2009). Both models present with inherent challenges that may obscure study results and conclusions. For example, some established sarcoma cell lines have been passaged for more than a decade which has been documented to influence both mutation rates and genetic stability (Sherr and DePinho, 2000). Further, immune compromised xenograft mouse models do not harbor an environment that accurately recapitulates the native *in vivo* sarcoma milieu. This is due to differing graft-host stromal interactions and the absence of a functional immune system (Pelleitier and Montplaisir, 1975) which have tremendous consequences on microenvironment interactions and tumor organizational architecture. Issues such as these may help to explain why numerous promising EWS therapeutic agents identified in preclinical investigations have been slow to progress to clinical development [i.e., targeted inhibitors of EWS-FLI1 activity: YK-4-279 (Erkizan et al., 2009; Barber-Rotenberg et al., 2012) and mithramycin (Grohar et al., 2011)] or unsuccessfully reproduce anticipated salubrious effects in early phase clinical trials (i.e., IGF1R blocking antibodies: R1507 (Pappo et al., 2014), cixutumumab (Malempati et al., 2012), and figitumumab (Juergens et al., 2011). Based on the results of this study, EWS-iPS cells may constitute a novel platform to bypass the aforementioned difficulties associated with the establishment and expansion of primary cell lines from patient tumors. This may also afford the ability to expand primary cells that fail to grow under standard *in vitro* conditions—forgoing the need for xenotransplantation in *in vivo* models. Following reprogramming, EWS-iPS cells can be expanded limitlessly and routinely re-differentiated into characteristic Ewing cells for high throughput drug screens and/or other analyses requiring large quantities of viable cells.

EWS appears to exhibit a clinical profile whose underlying biology is consistent with the cancer stem cell model. In this model, disease relapse is attributed to the survival and expansion of a subpopulation of cancer stem cells (CSCs) exhibiting a cytotoxic drug resistant phenotype. Prospective isolation of CSCs has previously been reported in EWS where high ALDH expressing, chemotherapy-resistant stem-like subpopulations were isolated from related cell lines and human xenografts (Awad et al., 2010). These EWS CSCs expressed putative pluripotency transcripts (i.e., OCT4 and NANOG), exhibited tumor initiating activity *in vivo*, and possessed a chemotherapy-resistant phenotype. In this report, EWS CSCs appeared to share a number of phenotypic properties with EWS-iPS cells, although EWS CSCs maintained sensitivity to the pharmacological EWS-FLI1 inhibitor, YK-4-279, implicating sustained maintenance of EWS-FLI1 oncogene addiction or rather a dependency on EWS-FLI1 signaling. Unlike EWS CSCs (Awad et al., 2010), EWS-iPS cells acquired resistance to agents targeting EWS-FLI1, i.e., mithramycin and YK-4-279. This suggests that epigenetic modifications during the reprogramming process or activation of specific pathways required for iPS cell maintenance may supersede downstream EWS-FLI1-mediated pathways. Given that there exists subpopulations of EWS CSCs that exhibit heightened expression of pluripotency associated transcripts it is tempting to speculate that there may exist a portion of cells exhibiting a similar phenotype to that of EWS-iPS cells. Losing the requirement for EWS-FLI1 oncogene signaling could explain, in part, the acquisition of resistance to IGF1R inhibitors in EWS patients, a major downstream effector pathway of EWS-FLI1 and significant contributor to the oncogenic phenotype in EWS. The expression of the EWS-FLI1 fusion gene in EWS induces post-transcriptional derepression of IGF pathway components (Prieur et al., 2004; McKinsey et al., 2011) which consequently enhances MAPK/ERK as well as PI3K/AKT/mTOR signaling (Silvany et al., 2000; Zenali et al., 2009). Analogous to what was observed with agents that target EWS-FLI1 activity, EWS-iPS cells appeared to be largely refractory to small molecule inhibitors targeting putative downstream effector, signaling pathways of EWS-FLI1, i.e., ERK1/2, mTOR, and PI3K. As both ERK1/2 and AKT phosphorylation have been suggested to play important roles in the maintenance of iPS and ES cell lines (Brill et al., 2009; Wang et al., 2009) via an interplay with pluripotency transcriptional network, we postulate that the signaling for iPS cell maintenance likely compensates for direct inhibition of EWS-FLI1 and/or inhibition of its downstream effector pathways; a phenomenon that could hold true and, in part, underlie an additional mode of chemotherapeutic resistance in a minor subpopulation of EWS cells. Thus, given the high mortality rate and the possible cancer stem cell involvement in metastasis and tumor recurrence (Awad et al., 2010), undifferentiated EWS-iPS cells themselves may also provide a valuable tool to investigate EWS tumor development and modes of drug resistance via identifying the epigenetic modifications and molecular pathway alterations contributing to their drug resistance—current avenues of research that we are pursuing.

### Conflict of interest statement

The authors declare that the research was conducted in the absence of any commercial or financial relationships that could be construed as a potential conflict of interest.

## References

[B1] Arvand A. Denny C. T. (2001). Biology of EWS/ETS fusions in Ewing's family tumors. Oncogene 20, 5747–5754. 10.1038/sj.onc.1204598 11607824

[B2] Awad O. Yustein J. T. Shah P. Gul N. Katuri V. O'Neill A.. (2010). High ALDH activity identifies chemotherapy-resistant Ewing's sarcoma stem cells that retain sensitivity to EWS-FLI1 inhibition. PLoS ONE. 5:e13943. 10.1371/journal.pone.0013943 21085683 PMC2978678

[B3] Barber-Rotenberg J. S. Selvanathan S. P. Kong Y. Erkizan H. V. Snyder T. M. Hong S. P.. (2012). Single enantiomer of YK-4-279 demonstrates specificity in targeting the oncogene EWS-FLI1. Oncotarget 3, 172–182. 22383402 10.18632/oncotarget.454 PMC3326647

[B4] Braunreiter C. L. Hancock J. D. Coffin C. M. Boucher K. M. Lessnick S. L. (2006). Expression of EWS-ETS fusions in NIH3T3 cells reveals significant differences to Ewing's sarcoma. Cell Cycle 5, 2753–2759. 10.4161/cc.5.23.3505 17172842

[B5] Brill L. M. Xiong W. Lee K. B. Ficarro S. B. Crain A. Xu Y.. (2009). Phosphoproteomic analysis of human embryonic stem cells. Cell Stem Cell 5, 204–213. 10.1016/j.stem.2009.06.002 19664994 PMC2726933

[B6] Brown H. F. Unger C. Whitehouse A. (2013). Potential of herpesvirus saimiri-based vectors to reprogram a somatic Ewing's sarcoma family tumor cell line. J. Virol. 87, 7127–7139. 10.1128/JVI.03147-12 23596304 PMC3676103

[B7] Burchill S. A. (2003). Ewing's sarcoma: diagnostic, prognostic, and therapeutic implications of molecular abnormalities. J. Clin. Pathol. 56, 96–102. 10.1136/jcp.56.2.96 12560386 PMC1769883

[B8] Carette J. E. Pruszak J. Varadarajan M. Blomen V. A. Gokhale S. Camargo F. D.. (2010). Generation of iPSCs from cultured human malignant cells. Blood 115, 4039–4042. 10.1182/blood-2009-07-231845 20233975 PMC2875096

[B9] Chou B. K. Mali P. Huang X. Ye Z. Dowey S. N. Resar L. M.. (2011). Efficient human iPS cell derivation by a non-integrating plasmid from blood cells with unique epigenetic and gene expression signatures. Cell Res. 21, 518–529. 10.1038/cr.2011.12 21243013 PMC3193421

[B10] Dini G. Funghini S. Witort E. Magnelli L. Fanti E. Rifkin D. B.. (2002). Overexpression of the 18 kDa and 22/24 kDa FGF-2 isoforms results in differential drug resistance and amplification potential. J. Cell. Physiol. 193, 64–72. 10.1002/jcp.10152 12209881

[B11] Erkizan H. V. Kong Y. Merchant M. Schlottmann S. Barber-Rotenberg J. S. Yuan L.. (2009). A small molecule blocking oncogenic protein EWS-FLI1 interaction with RNA helicase A inhibits growth of Ewing's sarcoma. Nat. Med. 15, 750–756. 10.1038/nm.1983 19584866 PMC2777681

[B12] Grier H. E. Krailo M. D. Tarbell N. J. Link M. P. Fryer C. J. Pritchard D. J.. (2003). Addition of ifosfamide and etoposide to standard chemotherapy for Ewing's sarcoma and primitive neuroectodermal tumor of bone. N. Engl. J. Med. 348, 694–701. 10.1056/NEJMoa020890 12594313

[B13] Grohar P. J. Woldemichael G. M. Griffin L. B. Mendoza A. Chen Q. R. Yeung C.. (2011). Identification of an inhibitor of the EWS-FLI1 oncogenic transcription factor by high-throughput screening. J. Natl. Cancer Inst. 103, 962–978. 10.1093/jnci/djr156 21653923 PMC3119649

[B14] Hancock J. D. Lessnick S. L. (2008). A transcriptional profiling meta-analysis reveals a core EWS-FLI gene expression signature. Cell Cycle 7, 250–256. 10.4161/cc.7.2.5229 18256529

[B15] Hochedlinger K. Blelloch R. Brennan C. Yamada Y. Kim M. Chin L.. (2004). Reprogramming of a melanoma genome by nuclear transplantation. Genes Dev. 18, 1875–1885. 10.1101/gad.1213504 15289459 PMC517407

[B16] Hotta A. Cheung A. Y. Farra N. Garcha K. Chang W. Y. Pasceri P.. (2009). EOS lentiviral vector selection system for human induced pluripotent stem cells. Nat. Protoc. 4, 1828–1844. 10.1038/nprot.2009.201 20010937

[B17] Hu-Lieskovan S. Zhang J. Wu L. Shimada H. Schofield D. E. Triche T. J. (2005). EWS-FLI1 fusion protein up-regulates critical genes in neural crest development and is responsible for the observed phenotype of Ewing's family of tumors. Cancer Res. 65, 4633–4644. 10.1158/0008-5472.CAN-04-2857 15930281

[B18] Juergens H. Daw N. C. Geoerger B. Ferrari S. Villarroel M. Aerts I.. (2011). Preliminary efficacy of the anti-insulin-like growth factor type 1 receptor antibody figitumumab in patients with refractory Ewing sarcoma. J. Clin. Oncol. 29, 4534–4540. 10.1200/JCO.2010.33.0670 22025154 PMC3236653

[B19] Kumano K. Arai S. Hosoi M. Taoka K. Takayama N. Otsu M.. (2012). Generation of induced pluripotent stem cells from primary chronic myelogenous leukemia patient samples. Blood 119, 6234–6242. 10.1182/blood-2011-07-367441 22592606

[B20] Lessnick S. L. Dacwag C. S. Golub T. R. (2002). The Ewing's sarcoma oncoprotein EWS/FLI induces a p53-dependent growth arrest in primary human fibroblasts. Cancer Cell 1, 393–401. 10.1016/S1535-6108(02)00056-9 12086853

[B21] Lessnick S. L. Ladanyi M. (2012). Molecular pathogenesis of Ewing sarcoma: new therapeutic and transcriptional targets. Annu. Rev. Pathol. 7, 145–159. 10.1146/annurev-pathol-011110-130237 21942527 PMC3555146

[B22] Malempati S. Weigel B. Ingle A. M. Ahern C. H. Carroll J. M. Roberts C. T.. (2012). Phase I/II trial and pharmacokinetic study of cixutumumab in pediatric patients with refractory solid tumors and Ewing sarcoma: a report from the Children's Oncology Group. J. Clin. Oncol. 30, 256–262. 10.1200/JCO.2011.37.4355 22184397 PMC3269952

[B23] May W. A. Arvand A. Thompson A. D. Braun B. S. Wright M. Denny C. T. (1997). EWS/FLI1-induced manic fringe renders NIH 3T3 cells tumorigenic. Nat. Genet. 17, 495–497. 10.1038/ng1297-495 9398859

[B24] May W. A. Gishizky M. L. Lessnick S. L. Lunsford L. B. Lewis B. C. Delattre O.. (1993a). Ewing sarcoma 11;22 translocation produces a chimeric transcription factor that requires the DNA-binding domain encoded by FLI1 for transformation. Proc. Natl. Acad. Sci. U.S.A. 90, 5752–5756. 10.1073/pnas.90.12.5752 8516324 PMC46800

[B25] May W. A. Grigoryan R. S. Keshelava N. Cabral D. J. Christensen L. L. Jenabi J.. (2013). Characterization and drug resistance patterns of Ewing's sarcoma family tumor cell lines. PLoS ONE 8:e80060. 10.1371/journal.pone.0080060 24312454 PMC3846563

[B26] May W. A. Lessnick S. L. Braun B. S. Klemsz M. Lewis B. C. Lunsford L. B.. (1993b). The Ewing's sarcoma EWS/FLI-1 fusion gene encodes a more potent transcriptional activator and is a more powerful transforming gene than FLI-1. Mol. Cell Biol. 13, 7393–7398. 8246959 10.1128/mcb.13.12.7393-7398.1993 PMC364810

[B27] McKinsey E. L. Parrish J. K. Irwin A. E. Niemeyer B. F. Kern H. B. Birks D. K.. (2011). A novel oncogenic mechanism in Ewing sarcoma involving IGF pathway targeting by EWS/Fli1-regulated microRNAs. Oncogene 30, 4910–4920. 10.1038/onc.2011.197 21643012 PMC4696862

[B28] Mills J. Matos T. Charytonowicz E. Hricik T. Castillo-Martin M. Remotti F.. (2009). Characterization and comparison of the properties of sarcoma cell lines *in vitro* and *in vivo*. Hum. Cell 22, 85–93. 10.1111/j.1749-0774.2009.00073.x 19874397 PMC3000410

[B29] Miser J. S. Goldsby R. E. Chen Z. Krailo M. D. Tarbell N. J. Link M. P.. (2007). Treatment of metastatic Ewing sarcoma/primitive neuroectodermal tumor of bone: evaluation of increasing the dose intensity of chemotherapy–a report from the Children's Oncology Group. Pediatr. Blood Cancer 49, 894–900. 10.1002/pbc.21233 17584910

[B30] Miyoshi N. Ishii H. Nagai K. Hoshino H. Mimori K. Tanaka F.. (2010). Defined factors induce reprogramming of gastrointestinal cancer cells. Proc. Natl. Acad. Sci. U.S.A. 107, 40–45. 10.1073/pnas.0912407107 20018687 PMC2806714

[B31] Nishino K. Toyoda M. Yamazaki-Inoue M. Fukawatase Y. Chikazawa E. Sakaguchi H.. (2011). DNA methylation dynamics in human induced pluripotent stem cells over time. PLoS Genet. 7:e1002085. 10.1371/journal.pgen.1002085 21637780 PMC3102737

[B32] Okada M. Yoneda Y. (2011). The timing of retroviral silencing correlates with the quality of induced pluripotent stem cell lines. Biochim. Biophys. Acta 1810, 226–235. 10.1016/j.bbagen.2010.10.004 20965232

[B33] Pappo A. S. Vassal G. Crowley J. J. Bolejack V. Hogendoorn P. C. Chugh R.. (2014). A phase 2 trial of R1507, a monoclonal antibody to the insulin-like growth factor-1 receptor (IGF-1R), in patients with recurrent or refractory rhabdomyosarcoma, osteosarcoma, synovial sarcoma, and other soft tissue sarcomas: results of a sarcoma alliance for research through collaboration study. Cancer 120, 2448–2456. 10.1002/cncr.28728 24797726 PMC7009782

[B34] Park I. H. Zhao R. West J. A. Yabuuchi A. Huo H. Ince T. A.. (2008). Reprogramming of human somatic cells to pluripotency with defined factors. Nature 451, 141–146. 10.1038/nature06534 18157115

[B35] Pelleitier M. Montplaisir S. (1975). The nude mouse: a model of deficient T-cell function. Methods Achiev. Exp. Pathol. 7, 149–166. 1105061

[B36] Prieur A. Tirode F. Cohen P. Delattre O. (2004). EWS/FLI-1 silencing and gene profiling of Ewing cells reveal downstream oncogenic pathways and a crucial role for repression of insulin-like growth factor binding protein 3. Mol. Cell. Biol. 24, 7275–7283 10.1128/MCB.24.16.7275-7283.2004 15282325 PMC479730

[B37] Ries L. A. G. SEER Program (National Cancer Institute (US)). (1999). Cancer Incidence and Survival among Children and Adolescents: United States SEER Program 1975–1995. Bethesda, MD: National Cancer Institute, SEER Program, 1–182.

[B38] Riggi N. Suva M. L. Suva D. Cironi L. Provero P. Tercier S.. (2008). EWS-FLI-1 expression triggers a Ewing's sarcoma initiation program in primary human mesenchymal stem cells. Cancer Res. 68, 2176–2185. 10.1158/0008-5472.CAN-07-1761 18381423

[B39] Rorie C. J. Thomas V. D. Chen P. Pierce H. H. O'Bryan J. P. Weissman B. E. (2004). The Ews/Fli-1 fusion gene switches the differentiation program of neuroblastomas to Ewing sarcoma/peripheral primitive neuroectodermal tumors. Cancer Res. 64, 1266–1277. 10.1158/0008-5472.CAN-03-3274 14973077

[B40] Scotlandi K. Benini S. Nanni P. Lollini P. L. Nicoletti G. Landuzzi L.. (1998). Blockage of insulin-like growth factor-I receptor inhibits the growth of Ewing's sarcoma in athymic mice. Cancer Res. 58, 4127–4131. 9751624

[B41] Sherr C. J. DePinho R. A. (2000). Cellular senescence: mitotic clock or culture shock? Cell 102, 407–410. 10.1016/S0092-8674(00)00046-5 10966103

[B42] Silvany R. E. Eliazer S. Wolff N. C. Ilaria R. L. Jr. (2000). Interference with the constitutive activation of ERK1 and ERK2 impairs EWS/FLI-1- dependent transformation. Oncogene 19, 4523–4530. 10.1038/sj.onc.1203811 11002425

[B43] Takahashi K. Tanabe K. Ohnuki M. Narita M. Ichisaka T. Tomoda K.. (2007). Induction of pluripotent stem cells from adult human fibroblasts by defined factors. Cell 131, 861–872. 10.1016/j.cell.2007.11.019 18035408

[B44] Utikal J. Maherali N. Kulalert W. Hochedlinger K. (2009). Sox2 is dispensable for the reprogramming of melanocytes and melanoma cells into induced pluripotent stem cells. J. Cell Sci. 122, 3502–3510. 10.1242/jcs.054783 19723802 PMC2746132

[B45] Vormoor J. Baersch G. Decker S. Hotfilder M. Schafer K. L. Pelken L.. (2001). Establishment of an *in vivo* model for pediatric Ewing tumors by transplantation into NOD/scid mice. Pediatr. Res. 49, 332–341. 10.1203/00006450-200103000-00006 11228258

[B46] Wang X. Lin G. Martins-Taylor K. Zeng H. Xu R. H. (2009). Inhibition of caspase-mediated anoikis is critical for basic fibroblast growth factor-sustained culture of human pluripotent stem cells. J. Biol. Chem. 284, 34054–34064 10.1074/jbc.M109.052290 19828453 PMC2797176

[B47] Yu J. Hu K. Smuga-Otto K. Tian S. Stewart R. Slukvin I. I.. (2009). Human induced pluripotent stem cells free of vector and transgene sequences. Science 324, 797–801. 10.1126/science.1172482 19325077 PMC2758053

[B48] Zenali M. J. Zhang P. L. Bendel A. E. Brown R. E. (2009). Morphoproteomic confirmation of constitutively activated mTOR, ERK, and NF-kappaB pathways in Ewing family of tumors. Ann. Clin. Lab. Sci. 39, 160–166. 19429803

[B49] Ziegler K. Bui T. Frisque R. J. Grandinetti A. Nerurkar V. R. (2004). A rapid in vitro polyomavirus DNA replication assay. J. Virol. Methods 122, 123–127. 10.1016/j.jviromet.2004.08.012 15488630

